# Trend and principal components of HIV/AIDS among adults in SSA

**DOI:** 10.1038/s41598-024-55872-2

**Published:** 2024-05-15

**Authors:** Bayuh Asmamaw Hailu

**Affiliations:** Monitoring and Evaluation, Wollo University, Dessie, Ethiopia

**Keywords:** Diseases, Health care, Risk factors

## Abstract

This study aimed to identify the most important principal components (PCs) that contribute to the prevalence and change of HIV/AIDS in 44 SSA and data from different national and international datasets. The study estimated HIV prevalence, trend, and principal component analysis (PCA). Using the elbow method, the number of important PCs and contributions was identified. The quality of representation was checked, and more contributing variables for most important PCs were identified. Finally, the status by prevalence, the progress by trend, the more influenced component by PCA, and the more influenced variable with quality of representation by PCs were reported. The study found that HIV prevalence varied significantly, with 30 of the countries showed good progress/decline. Four PCs accounted for 51% of the total variance. Literacy, cohabitation, media exposure, and HIV status awareness are highly contributing factors. Based on these findings, a gap-based response will help reduce the burden of HIV.

## Introduction

Since the beginning of the first decade of the twentieth century, there have been several landmark events in global efforts to advance HIV/AIDS response. These include the Ryan White Comprehensive AIDS Resources Emergency in 1990^1^, the red ribbon symbol of AIDS in 1991^2^, the establishment of The Joint United Nations Programme on HIV/AIDS in 1996^3^, antiretroviral therapy was introduced in 1996^4^, goal was set to halt the spread of HIV/AIDS by 2015^5^, the President’s Emergency Plan for AIDS Relief was launched in 2003^6^.

In 2015, the world delivered on the AIDS targets of Millennium Development Goal 6—halting and ensuring healthy lives^7^ and promoting well-being for all at all ages^8^. In 2016, The United Nations General Assembly adopted the Political Declaration on Ending AIDS, which set new targets for 2020 and 2030^9^. In 2018, UNAIDS launched the “Miles to go—closing gaps, breaking barriers, righting injustices” report, which highlighted the progress made in the global response to HIV/AIDS and identified areas where more work is needed^10^. In 2019, the World Health Organization (WHO) recommended that people at high risk of HIV infection should be offered pre-exposure prophylaxis^11^. In 2020 and 2021, there were many long efforts to end the HIV epidemic globally and domestically^12^. However, in 2022 UNAIDS said that we are “IN DANGER”^13^.

Globally, HIV infection has declined annually from 2010 to 2022. However, around two-fifths of the region experienced an increase in HIV infection^14^. Sub-Saharan Africa (SSA) is home to two-thirds of all people living with HIV globally and is the hardest-hit region in the world^14^. While HIV varies across countries^15–17^ and within countries^18,19^, this heterogeneity has been explored to some extent. Each community contributes to a specific component, and the factors that influence each community more than their neighbors, the progress over time potential components and influenced variables on the most relevant components do not have been explored at all. This work clearly shows the prevalence, change over time, each community/variable contribution for the specific component, and the highly influenced variable in each component by community. Due to culture, economy, and other reasons, the way of response differs from one area to another area. Programmers, policymakers, and other concerned bodies can make more reduction efforts based on their problem. This analysis clearly showed what the progress is, where is the problem more happening and what the potential reason is clearly set.

## Methods

### Overview

The objective was to identify and obtain individual/community-level data from all repeated nationally representative Demography and Health Survey (DHS), Malaria Atlas Project, NASA Socioeconomic Data and Applications Center (SEDAC), Global Health Data Exchange (GHDx), and sentinel surveillance of antenatal care clinics (ANC data) that included information on HIV/AIDS status and/or other intervention areas. A maximum of 44 countries were included based on conditions, reducing some countries in some analyses. For example Zimbabwe included in the trend, PCA, community, and national prevalence. South Sudan, Mauritius, Equatorial Guinea, and Botswana included only in national prevalence analysis but not included in other analysis.

(Supplementary Figs. 1, 2).

For HIV analysis, data from DHS and GHDx were used^20,21^, which are highly comparable and nationally representative. DHS is one of the main publicly available sources of information for low-income and middle-income countries. Given their focus on adult HIV tests, these surveys target both women and men aged 15–49 years. SSA was selected because most of the countries have conducted at least one HIV test result or geospatial coordinate, and 15 of them collected data from 2015 to 2021 by DHS, almost all of them estimated by GHDx in 2018, and in 2021 by ANC sentinel^22^ (Supplementary Figs. 1–3; Supplementary Table 1). The ethical responsibility for all data sources included in this study lies with the institutions that conducted the surveys in each country; therefore, ethics approval for this study was not required.

### Data management and analysis

This analysis presents community-based prevalence from 2015 to 2021, country-level prevalence in 2021, and the change over time from 2000 to 2018 (some countries extended to 2021) in Sub-Saharan Africa. The period of 2000–2021 and the age group of 15–49 years were selected to maximize data availability, and most countries’ data sources focus on the 15–49 years age range as the most commonly reported age range. Trend in the form of regression ordinal logistic models were implemented. In this analysis, the data is converted into the prevalence of enumeration areas or communities but for this purpose converted to an ordinal model. Cluster-level HIV prevalence was recategorized into quartiles, where the first quarter is the lowest prevalence and the fourth quarter is the highest prevalence.

For Principal Component Analysis (PCA), only countries that had standard DHS data with GIS coordinate/shape file data information at least once since 2012 were included. Based on DHS coordinate data, extract the cluster value from other sources of data.

This analysis used community-based geographic coordinates (point) data rather than polygon (administrative areas) for HIV prevalence analysis. In point data, the value of HIV prevalence is shared country to country across the border. For this reason, the different years of data collection of each country could be influenced by other neighbors. To control the time variation of data collection, I adopted the country in which HIV test data was collected since 2015 from DHS and the rest from the 2018 GHDx. The country-level prevalence is data from sentinel surveillance of antenatal care clinics (ANC data). ANC data were primarily derived from national HIV estimate files developed by national teams and compiled and shared via UNAIDS. Therefore, I used final UNAIDS compiled data of 2021^22^. For change over time, used both DHS and GHDx data. For community-based change over time, I used GHDx raster data of 2000, 2005, 2010, and 2018. For country-based trend (change over time) regression analysis, all DHS datasets since 2000 which have HIV test results included were used. If the country did not have data or enough data within the 5 years of interval extracted from GDB raster data based on the most recent GPS coordinates of DHS (Supplementary Fig. 4).

This analysis included 27 pre-existing covariates from different sources, such as DHS^20^, Atlas Malaria project^23–25^, NASA socioeconomic data^26–31^, and GHDx^32^. Extracting the important information from multivariate data from different datasets to express this information as a set of few new important variables and the most important variables helps combat HIV and other similar problems to achieve 2030 goals. For this analysis, PCA was used. For PCA analysis, variables from DHS individual-level variables changed to community prevalence, and variables from other sources were extracted based on those community-based coordinate datasets. In general, 19,546 point data from 34 countries with 27 variables from four different datasets were extracted (Supplementary Fig. 2; Supplementary Table 1).

Due to the sensitivity of PCA for outliers, the outlier is identified using a boxplot. It is treated by replacing the outlier with a value that is close to the outlier but not as extreme (winsorization). If the outlier is extreme and cannot be treated by the winsorization method, first reduce the effect by using log transformation and finally use winsorization (Supplementary Fig. 3).

All variables are continuous but the effects were found in different dimensions. Some variables are disadvantaged by lower values, while others are inverse. To control this problem and put it into the same dimension (more negatively influenced by higher value), it is transformed by multiplying -1. The variables were from different sources and had different units of measurement. Due to this reason modifies by harmonizing its variability in all directions of the original vectors or standardizing the variables.

To obtain the results of PCA, the eigenvalues/variance and its percent obtained in each principal component are extracted and visualized. The eigenvalues are plotted according to their size (scree), and the elbow point is identified, which is the point where the slope of the graph goes from steep to flat. The components before the elbow are kept, which is determined by looking at an elbow shape of Scree Plot, which is the plot of eigenvalues ordered from largest to smallest^33–35^.

The quality of representation (cos^2^) of both variables and observations/clusters to each PCs were identified. The contributions of variables and clusters in accounting for the variability in a given PCs are expressed. The most significantly associated variables (according to their contributions) with a given principal component are identified by dimension description. All flow from data extraction to data analysis see the flow chart in the supplementary material (Supplementary Fig. 4).

This analysis do not use of experimental animals, or human participants and I use only global/national open source data. So, institutional and/or licensing committee approval for this study was not required.

Analyses were conducted using STAT for data management R for statistical computing (Outlier, PCA, graph, regression), and SAGA and QGIS (Interpolation).

### Ethical approval

The utilized DHS data sets are publicly available, and the DHS Programme de-identifies all data before making them available to the public. All other geospatial data sources and DHS do not contain variables at the level of human subjects. Therefore, this work did not require ethical approval.

## Results

**Figure 1:**
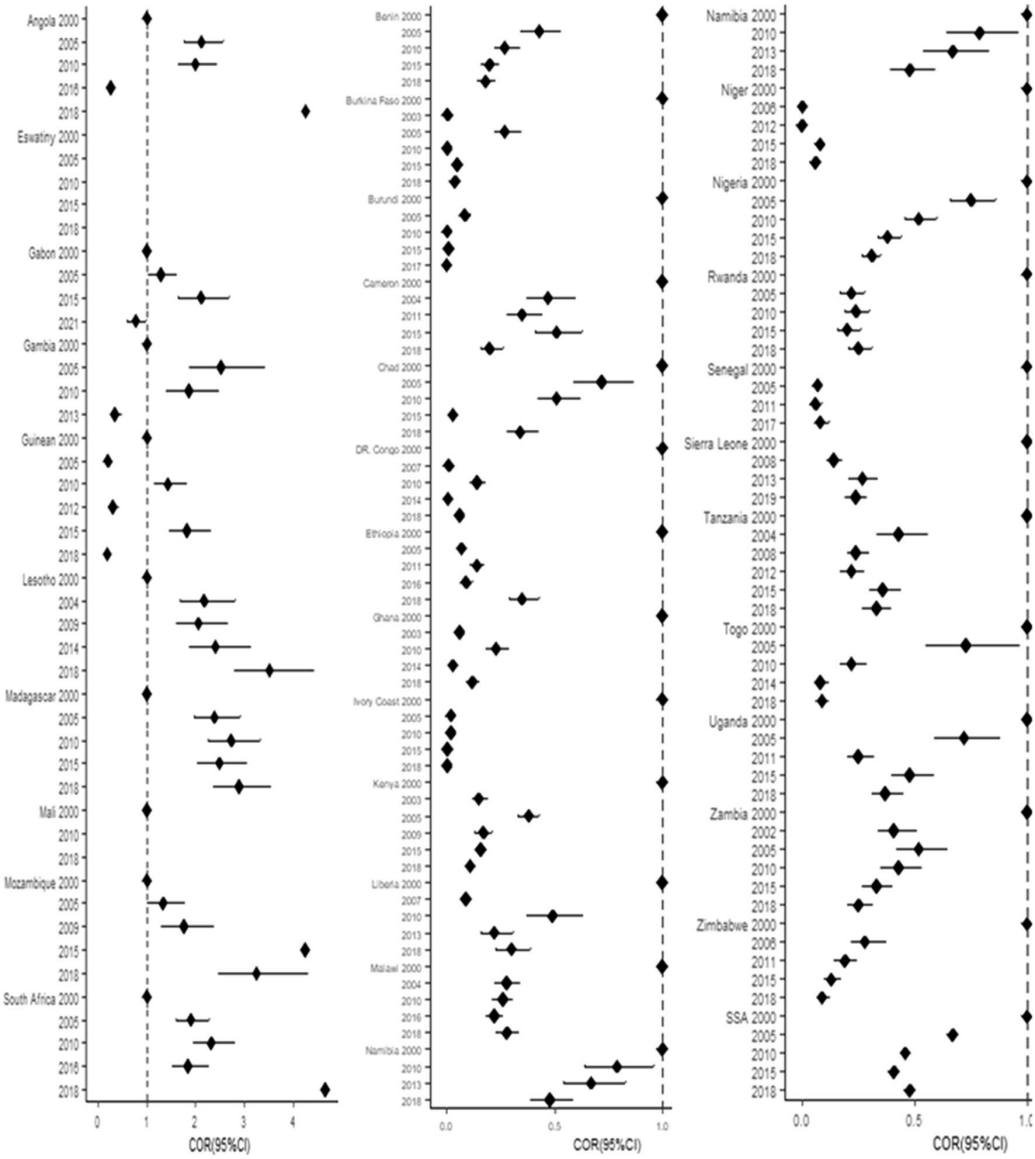
HIV trend in SSA. HIV change over time of each countries by year multilevel logistic regression compared by crude odds ratio (COR). To conduct this analysis use R version 4.3.2, available at: https://cran.r-project.org/bin/windows/base/.

HIV prevalence varied significantly at the community level and among countries throughout Sub-Saharan African countries. This variation indicated that every country had both edges of HIV prevalence within their communities or grid cells. Within countries, some had a relatively high overall HIV prevalence (e.g., Lesotho, Botswana, and South Africa). Out of 44 countries, except 10 countries have shown good progress or decline in HIV prevalence since 2000 (Fig. 1; Supplementary Figs. 5–7).

In Sub-Saharan Africa, HIV has declined the risk of transition to high severity from the beginning of the first decade of the twenty-first century to the end of the second decade of the twenty-first century. In most countries, decline occurred over time (e.g., Benin, Burkina Faso, Burundi), and in 10 countries it coincided with an increased risk of HIV moving from lower severity to higher severity in the recent time period (e.g., Angola, Lesotho). Some countries like Mali had lower prevalence; the trend indicates increased HIV severity since 2000. Some countries more infected by HIV like South Africa and Lesotho showed increased over time. Change over time based on each grid cell HIV prevalence in Sub-Saharan Africa showed that the change between the first decade of the twenty-first century at the beginning and the end had better achievement compared to the change between the second decade of at the beginning and the end. The southern part of Sub-Saharan Africa was a reverse achievement. Most countries continue as it has neither declined nor increased especially in the second decade of twenty-first century. The southern Sub-Saharan Africa in recent decades is good compared to the first decade but still showed a higher increment in recent decades (Fig. 1; Supplementary Figs. 5–7).

**Figure 2:**
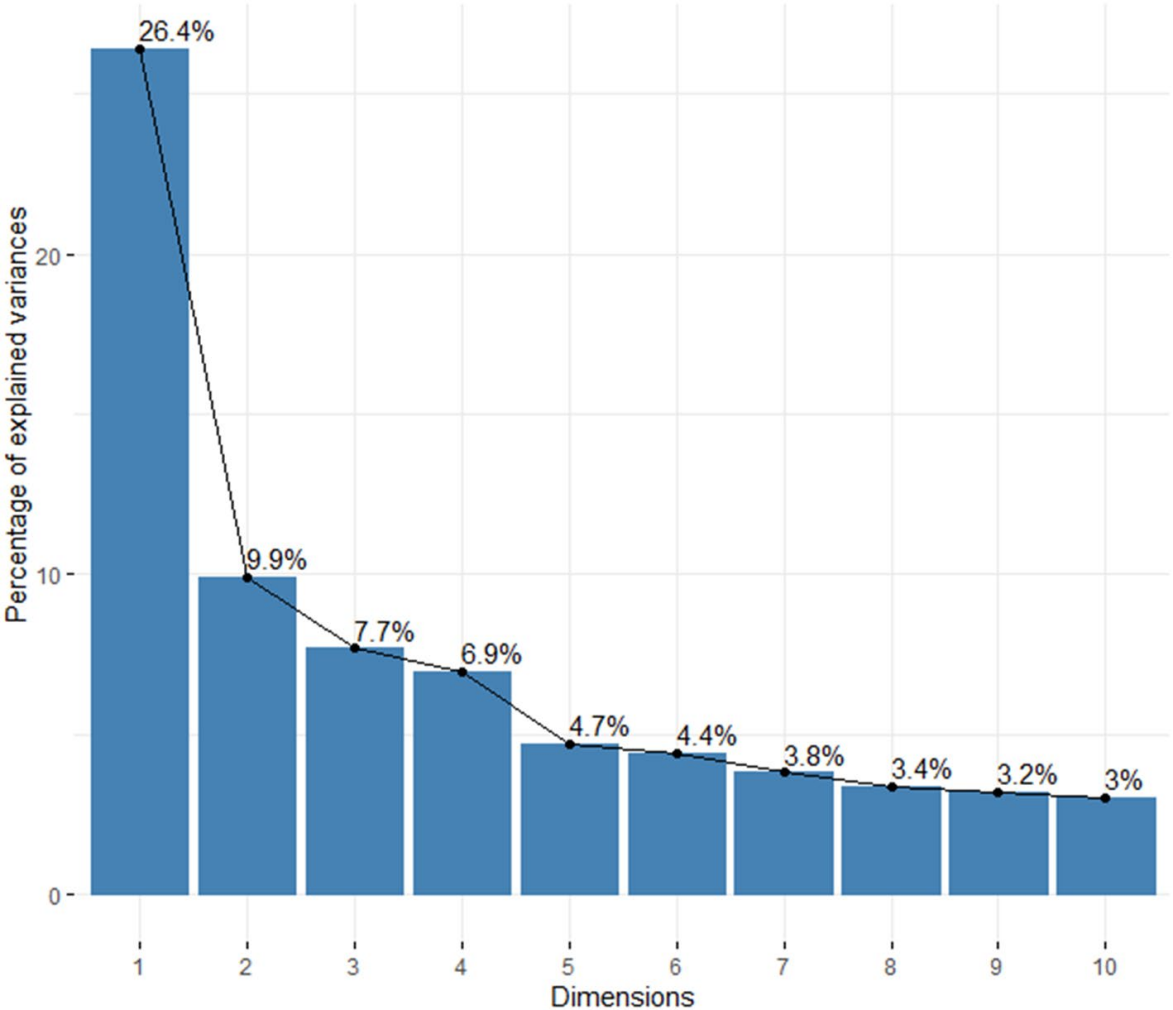
Eigenvalues/variances of principal components. Proportion of information retained by the top ten principal component.

The outcome measurement survey’s construct validity was calculated using PCA. According to PCA, the total of four principal components (PCs) could account for 51% of the total variance (based on the elbow method). The first component expresses 26.41% of data variability, which is 2.66, 3.44, and 3.8 times more variables than the next three components, respectively. The first two components represent 36.32% of the data (Fig. 2; Supplementary Table 2).

**Figure 3:**
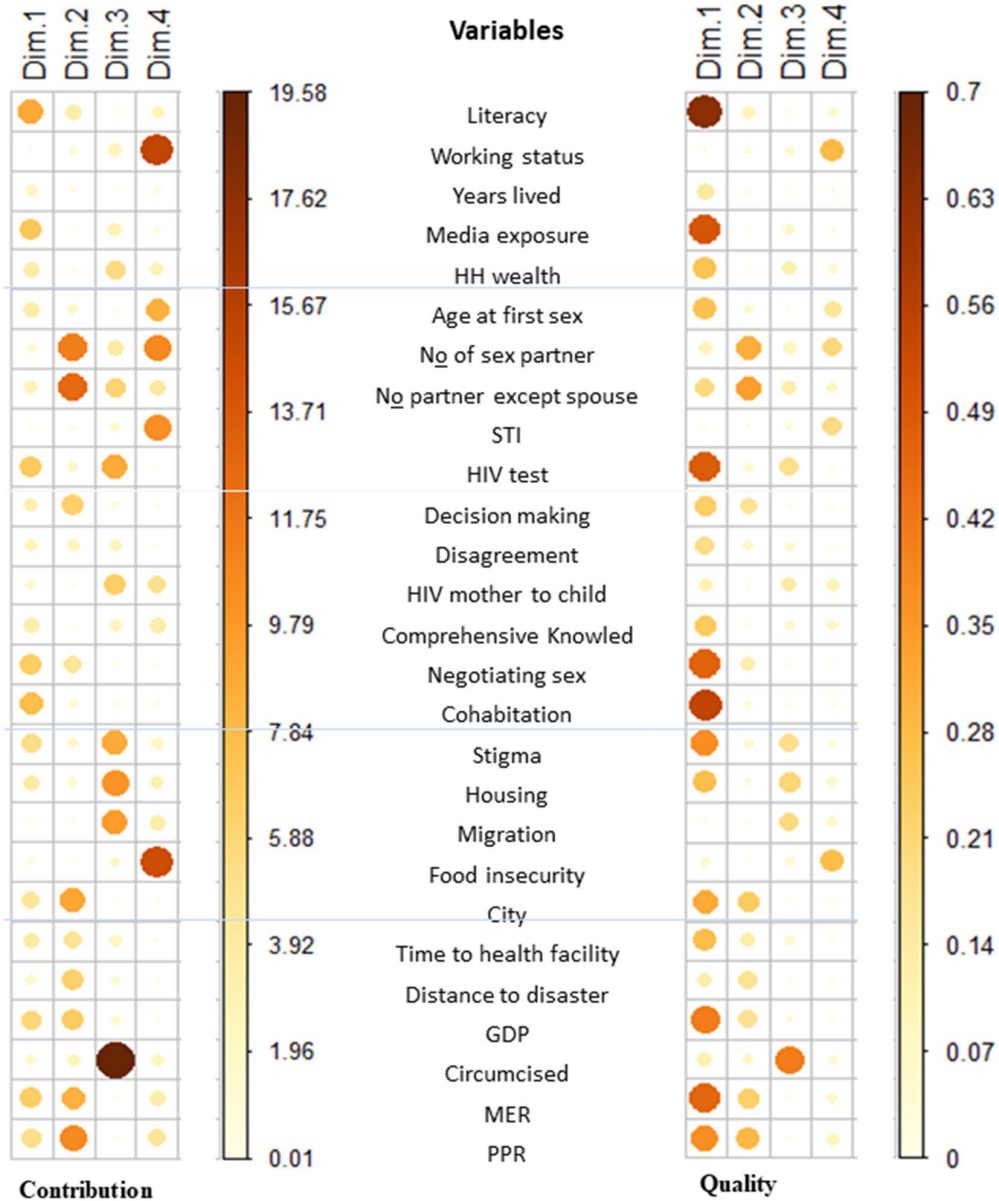
Variable contribution and Quality of representation for the selected first 4 PCs (Dim) in left and right panel respectively.

The list of variables that quality of representation was checked the most to the first or most influenced component were literacy, cohabitation, media exposure, HIV status, negotiating sex, MER, GDP, stigma, and PPT. These variables contributed 8.9%, 7.62%, 6.99%, 6.77%, 6.53%, 6.47%, 5.74%, 5.21%, and 5.16%, respectively. Literacy alone contributed 8.9%. In the second most influenced component, the number of partners excluding spouse, number of sex partners, PPT, city, MER, GDP, and disaster were the more contributing variables that accounted for 12.65%, 11.31%, 10.61%, 8.96%, 8.36%, 6.57%, and 6.36%. For the third more likely influenced component male circumcision, housing, migration, stigma, HIV test, and mother to child transmission knowledge were contributing variables that accounted for 19.58%, 10.05%, 9.57%, 8.77%, 8.7%, and 6.45% respectively. Working status (15.09%), food insecurity (14.54%), number of sex partners (10.74%), STI (10.33%), and age at first sex (8.34%) contributed to the fourth component. Those variables contributed about two thirds of the corresponding component (Fig. 3; Supplementary Figs. 8, 9; Supplementary Table 3).

The quality of representation of a variable in each principal component is well represented by the principal component. In every principal component separately, the high percentage of variable contributions has high quality of representation by principal components. Almost all the variables listed above contributed to the first component, and male circumcision in the third component was highly represented by its principal component (Fig. 3; Supplementary Fig. 10; Supplementary Table 4).

**Figure 4:**
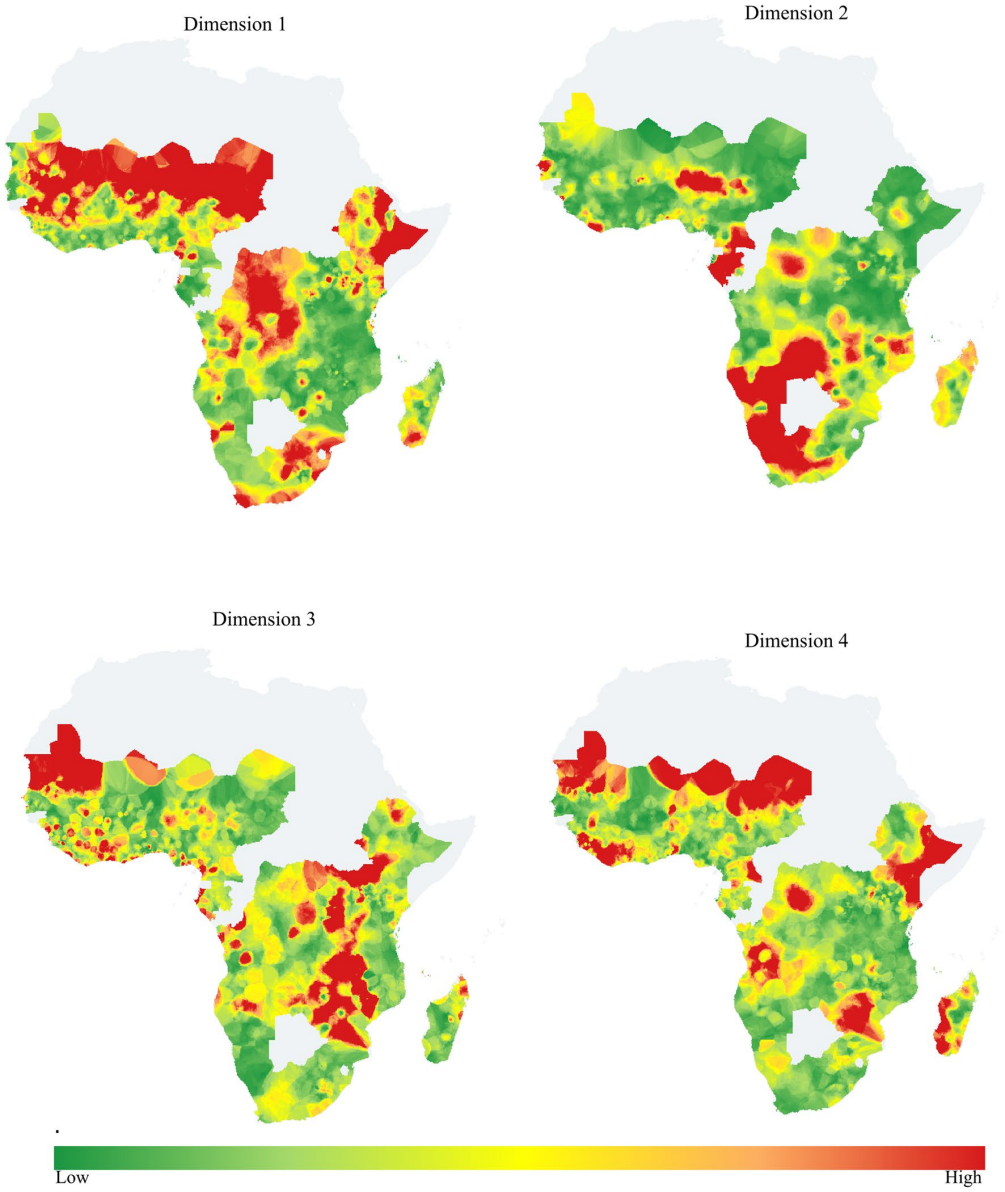
Communities’ contribution of for the selected first 4 PCs (Dimensions). To conduct this analysis use QGIS version 3.28.15 with SAGA GIS version 7.8.2, available at: https://download.qgis.org/downloads/.

The list of communities/clusters that contributed the most to the first or most influenced component were the Northern part of West Africa and most areas of DR Congo, Ethiopia, and South Africa. Communities from all areas of Gabon and Southern African countries except the North East direction of South Africa, some areas of Cameroon, Senegal, Nigeria, and Liberia were highly contributing to the second most principal components. The third most influenced components were highly contributed to by Mauritania, Rwanda, Burundi, countries in the southern part of East Africa and northern parts of South Africa, and some countries like Ivory Coast. In the fourth most influenced components, highly contributing communities were the Northern edge of West Africa, most areas of Sierra Leone, Liberia, Mozambique, Madagascar, Kenya and Ethiopia and some areas of other countries like Angola and DR Congo (Fig. 4).

The quality of representation of a cluster/community in each principal component is well represented by a principal component. In every principal component separately, the high percentage of community contributions has high quality of representation by principal components (Supplementary Fig. 11).

**Figure 5:**
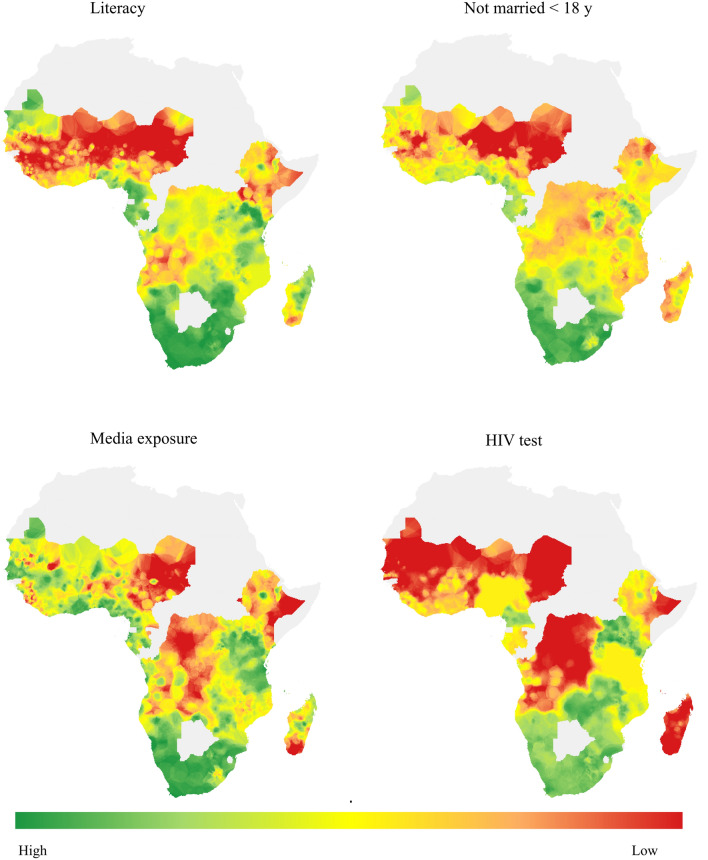
The prevalence of the most important variables highly contribute for the first most important component.

The four most significant variables contributing to the most important principal components were literacy, cohabitation, media exposure, and HIV status awareness. Most Western SSA and some Eastern SSA countries had a higher prevalence of communities unable to read and write. Except for southern SSA countries, the rest are living with the problem of being unable to read and write. The second most important variable was marital status. Most Western SSA countries of their community pass on unstable lives with partners. That means a higher percentage of the community is either divorced, widowed, or separated. The third important variable that contributed to the first principal components was media exposure. Most communities in central and some areas of Western and Eastern SSA did not have access to radio, television, and newsletters. The fourth was awareness of HIV status. Almost all countries in Western and Central SSA, Madagascar, and some areas of Ethiopia did not know their HIV status (Fig. 5).

**Figure 6:**
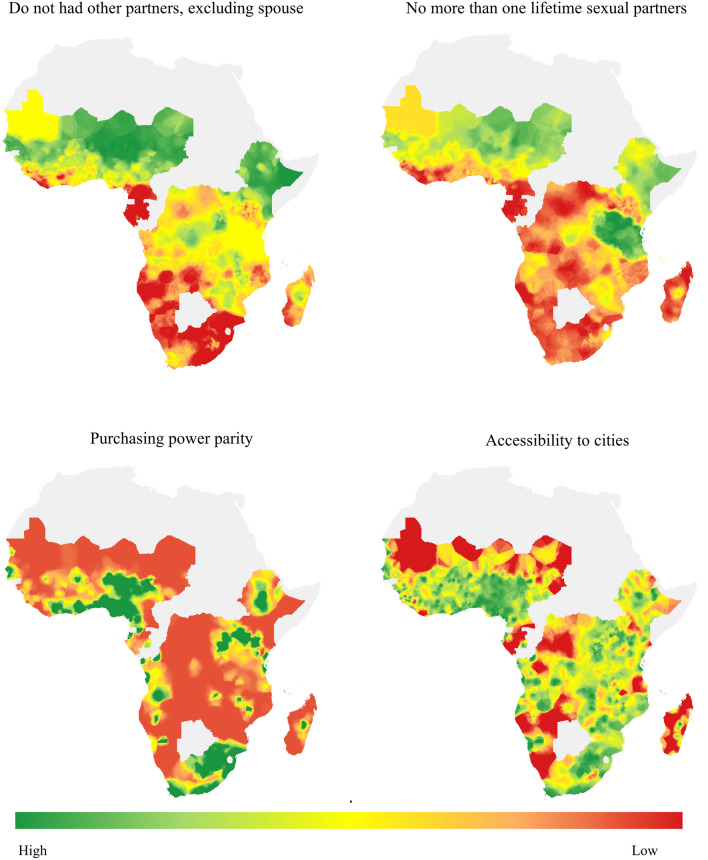
The prevalence of the most important variables for the second most important component.

The four most significant variables contributing to the second most important principal components were the number of partners excluding spouse, number of lifetime sexual partners, PPT, and accessibility cities. Almost all communities of Southern SSA, Gabon, Cameroon, and Liberia from Western SSA had at least one partner excluding a spouse. Most communities in all areas of Northern, Central, and Eastern SSA except Tanzania, Uganda, Ethiopia and Kenya in the direction of Ethiopia, Western edge of West Africa Gabon to Sera Lion had more and more number of sex partners. Except South Africa, Rwanda, Burundi, Uganda, Nigeria, and some areas of Benin, Togo, Ghana, Ivory Coast, Ethiopia, Kenya, and Angola were under the economic productivity and standards of living or lower productivity and growth. All areas of Mauritania, Chad, Madagascar, Namibia and Gabon, and some areas of Northern part of West Africa, D.R. Congo and others were talking a long time to travel time to cities to assess inequalities in accessibility (Fig. 6).

## Discussion

Since the first decade of the twenty-first century, the global community has been working to combat HIV and AIDS. The United Nations Security Council and the U.S. President’s Emergency Plan for AIDS Relief took different actions to respond to HIV and AIDS globally^36–38^. Thanks to an uninterrupted effort on access to antiretroviral therapy, HIV-positive people now live longer and healthier lives and prevent onward transmission, leading to a decline in HIV prevalence and death.

The global community has set interim targets for 2020 and 2025 to help achieve the goal of ending the AIDS epidemic by 2030. The 2020 targets for a declined number of newly infected and AIDS-related deaths were missed^39^, and this target is extended to 2025 with some improvement in reducing the burden^40,41^. Considering UNAIDS 2022^14^, achieving the 2025 target will be challenging. We are in the mid-year of the second interim target and now need to take more important action to achieve the target or reduce the gap. This work contributes to showing the path that ends AIDS. Not only does it contribute to HIV/AIDS efforts, but it also contributes to achieving other SDG goals by identifying present analysis and potential for future directions.

The proportion of HIV has declined over time in Sub-Saharan Africa (SSA) and fallen in most countries but increased in others. These analyses show substantial differences between and within countries in levels and trends in HIV prevalence and the spatial distribution of communities living with HIV. Some countries as well as their communities with high prevalence have increased since the beginning of the first decade of the twenty-first century^42^.

Most communities that contributed to PC1 were found in West Africa, D.R. Congo, Ethiopia, and the Northern part of South Africa. Most areas of West Africa had a higher prevalence of communities unable to read and write and did not know their HIV status. In East parts, there was an additional higher rate of being unable to stay in marriage and low access to media. Most areas of Central and Eastern SSA, especially D.R. Congo, Madagascar, and Ethiopia, had lower media exposure and awareness of HIV status. Efforts on those more significant variables in Western, Central, and Eastern SSA can reduce the gap of SDG goals and achievements not only in HIV/AIDS but also with other most SDG goals.

Most communities that contributed to PC2 were highly infected by HIV. In these areas, the community has more lifetime sexual partners even if they had partners exposed to more additional partners for sexual purposes. In SSA, reductions in the numbers of new HIV infections have been modest. Compared to other regions, SSA is home to new HIV infections^14^. For effective new HIV reduction working related to sexual intercourse is predominant. For HIV reduction hotspot of SSA there are the underline areas of intervention for tangible response were sexual-related problems.

This sexual-related problem not only in Southern SSA but also in Western SSA cannot be shown in response to PC1 Central Africa and Northern part of Eastern SSA including Madagascar. The other possible influencing factors in SSA were economic problems. Throughout all SSA countries except South Africa, some part of West Africa in the location of Ghana and its neighbor, and some part of East Africa showed a lack of economic growth and standards of living in different countries with a common currency/basket of goods and travel time to cities to assess inequalities in accessibility.

The diversity seen in permutations of levels of literacy, cohabitation, media access, HIV test, more sexual intercourse partners, and economic situation has led to considerable variations in ways in which different countries are addressing and meeting the targets. This analysis used for policy response can be used for effective HIV response. Additionally, this effort can be used for other SDG goal responses in parallel.

### Limitations

This analysis has several limitations. First, some SSA countries do not have data with DHS and are not included in this analysis. The data source is from different open-source datasets, and data collection time is varied. Some covariates of the surveys, potential non-response bias, and recall bias are particular concerns^43^. The covariate may also be suboptimal in some situations, such as age at first sex^44^. The surveys rely on self-reported data, which may be subject to recall bias or social desirability bias for covariates. Finally, the surveys may not be representative of certain subgroups of the population, such as those living in remote areas or those who are not included in the sampling frame^45^. Due to this reason, unmeasured communities are predicted by the result of the nearest neighbor community.

### Supplementary Information

Supplementary Information.

## Data Availability

The data that support the findings of this study are available from the DHS (http://www.measuredhs.com), NASA Socioeconomic Data and Applications Center (sedac) (https://sedac.ciesin.columbia.edu/), GBD/GHDx (https://ghdx.healthdata.org/ihme_data), Malaria Atlas Project (https://data.malariaatlas.org/trends?year=2020&metricGroup=Malaria&geographicLevel=admin0&metricSubcategory=Pf&metricType=rate&metricName=incidence), and UNAIDS (https://www.unaids.org/en/resources/documents/2021/2021_unaids_data). All data sources are publicly accessible except for the Department of Homeland Security (DHS), which necessitates a formal request. Therefore, data are available from the corresponding author (Bayuh Asmamaw Hailu) upon reasonable request.
