# Supplementary material for: Trend and principal components of HIV/AIDS among adults in SSA
Source: Sci Rep. 2024 May 15;14:11098. doi: 10.1038/s41598-024-55872-2 (PMC11096374; doi:10.1038/s41598-024-55872-2)
Supplement: Supplementary file 1 — Supplementary Information. [file 41598_2024_55872_MOESM1_ESM.docx]

**Supplementary information**

**Trends and specific intervention areas for combating HIV/AIDS in SSA: Multilevel and principal components**

Bayuh Asmamaw Hailu


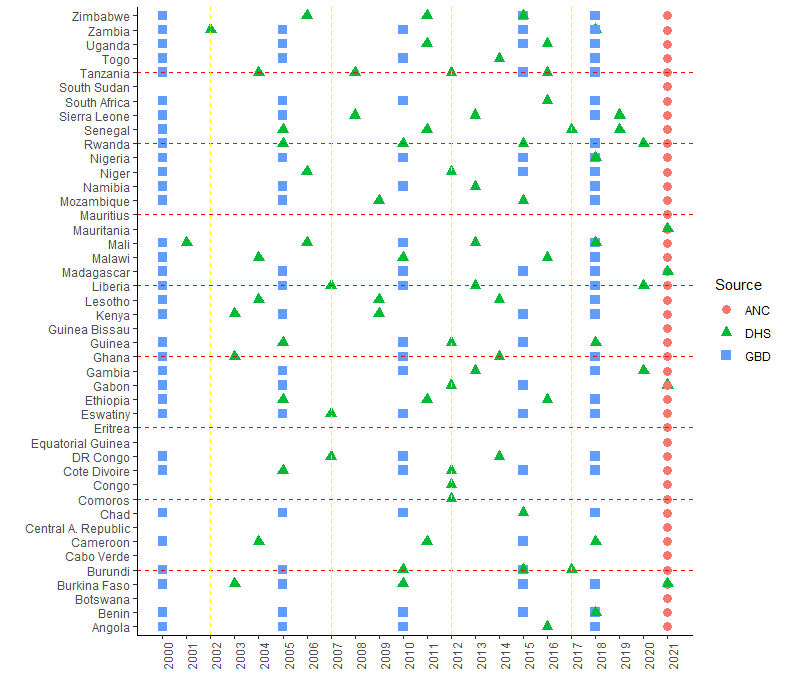


Supplementary Figure 1. Source of data by year in each country

Zimbabwe included in the trend, PCA, and community and national prevalence. South Sudan, Mauritius, Equatorial Guinea, and Botswana included only in national prevalence analysis but not included in other analysis.


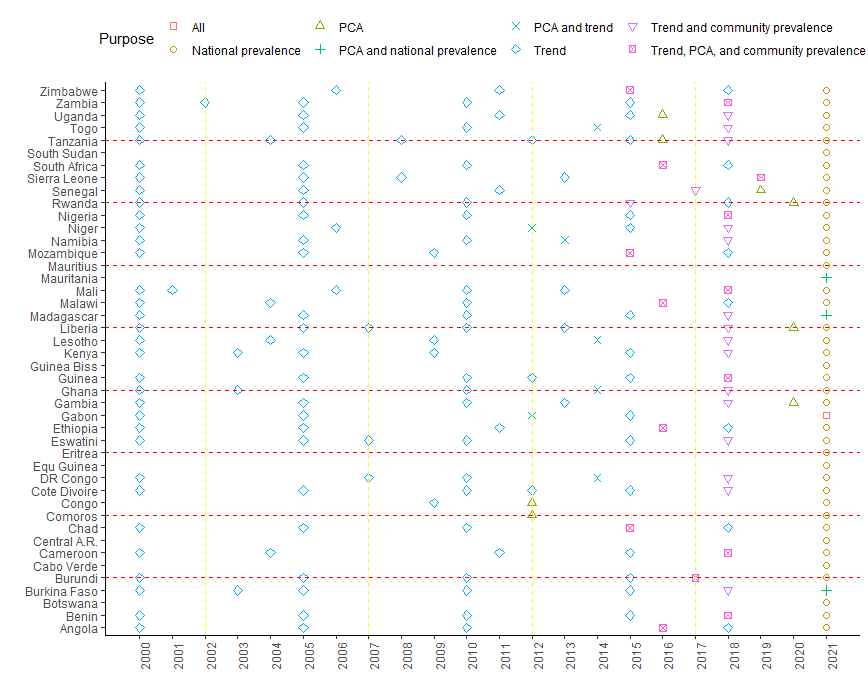


Supplementary Figure 2. The purpose of data to implement by year in each countries

Supplementary Table 1: List of covariates with source and description

| **Variable** | **Source** | **Description** |
| --- | --- | --- |
| City | Atlas malaria | Distance to city |
| Housing | Atlas malaria | Prevalence of improved housing |
| Time to health facility | Atlas malaria | Estimated travel time to nearest health facility |
| Circumcised | GBD | Prevalence of uncircumcised |
| MER | NASA | Market Exchange Rate |
| GDP | NASA | Gross Domestic Product |
| PPR/PPP | NASA | Purchasing Power Parity |
| Distance to disaster | NASA | Distance from the disaster |
| Food insecurity | NASA | Prevalence |
| Migration | NASA | Number of net migration |
| Literacy | DHS | Prevalence of cannot read at all |
| Cohabitation | DHS | Prevalence of previously married |
| Media exposure | DHS | Prevalence of do not have media access |
| HIV test | DHS | Prevalence of never tested for HIV |
| Negotiating sex | DHS | Prevalence of negotiating unsafe sex |
| Stigma | DHS | Prevalence of stigma |
| HH wealth | DHS | Prevalence of lower wealth |
| Age at first sex | DHS | Prevalence of first sex before 18th birth day |
| Comprehensive knowledge | DHS | Prevalence of do not have HIV knowledge |
| Decision making | DHS | Prevalence of women do not decision make |
| No partner except spouse | DHS | Prevalence of had at least one partner excluding spouse |
| Disagreement | DHS | Prevalence of women disagree with her husband |
| Years lived | DHS | Prevalence of living in the residence below 3 years |
| HIV mother to child | DHS | Prevalence of do not have transmission of mother to child knowledge |
| No of sex partner | DHS | Prevalence of number of sex partners above one |
| Working status | DHS | Prevalence of do not have work |
| STI | DHS | Prevalence of had any STI in the last 12 months |


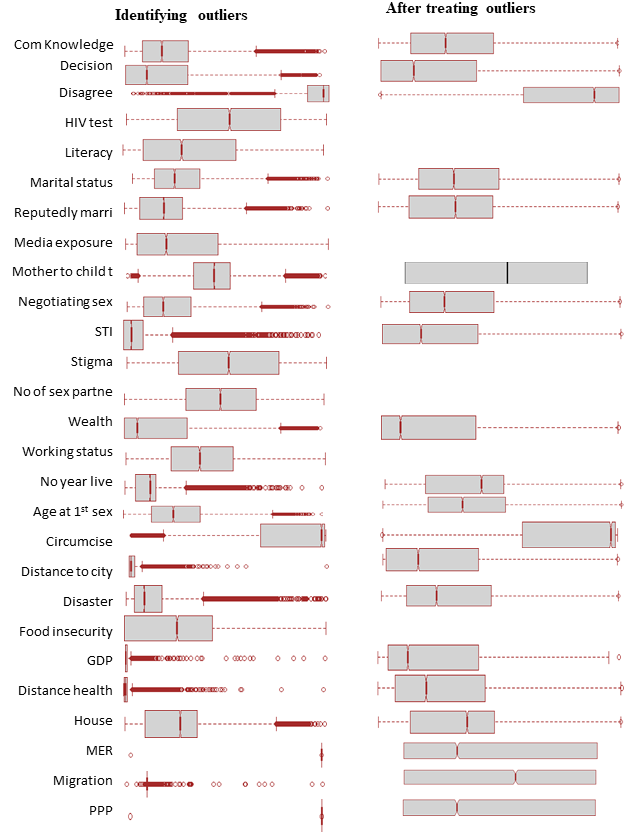


Supplementary Figure 3. Outlier identification and threat using winsorization method.

# **Analytic process**


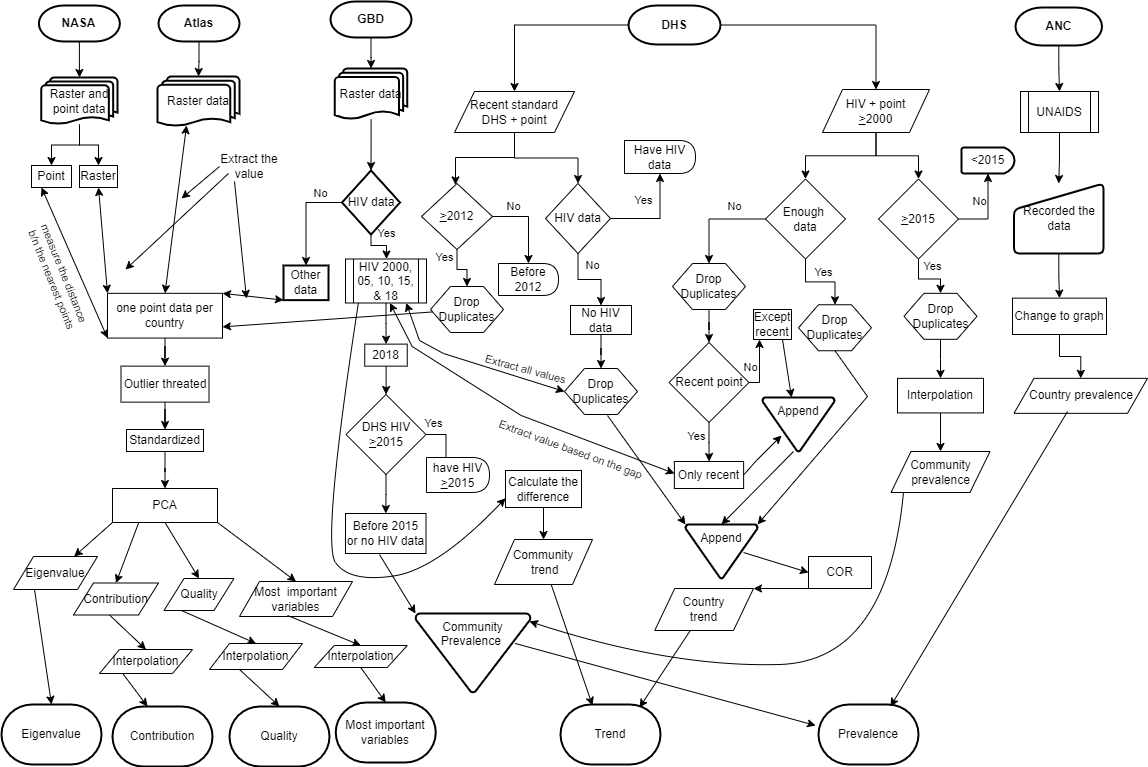


Supplementary Figure 4. Flow chart to show analytic process from start to end.


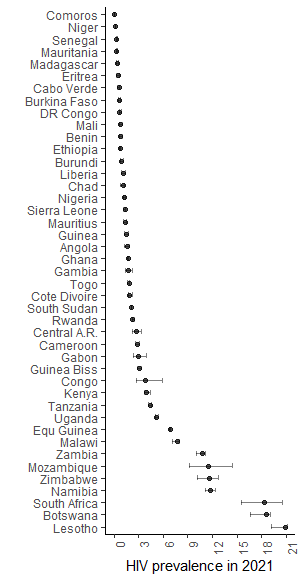


Supplementary Figure 5. HIV prevalence in SSA in each country in the year of 2021


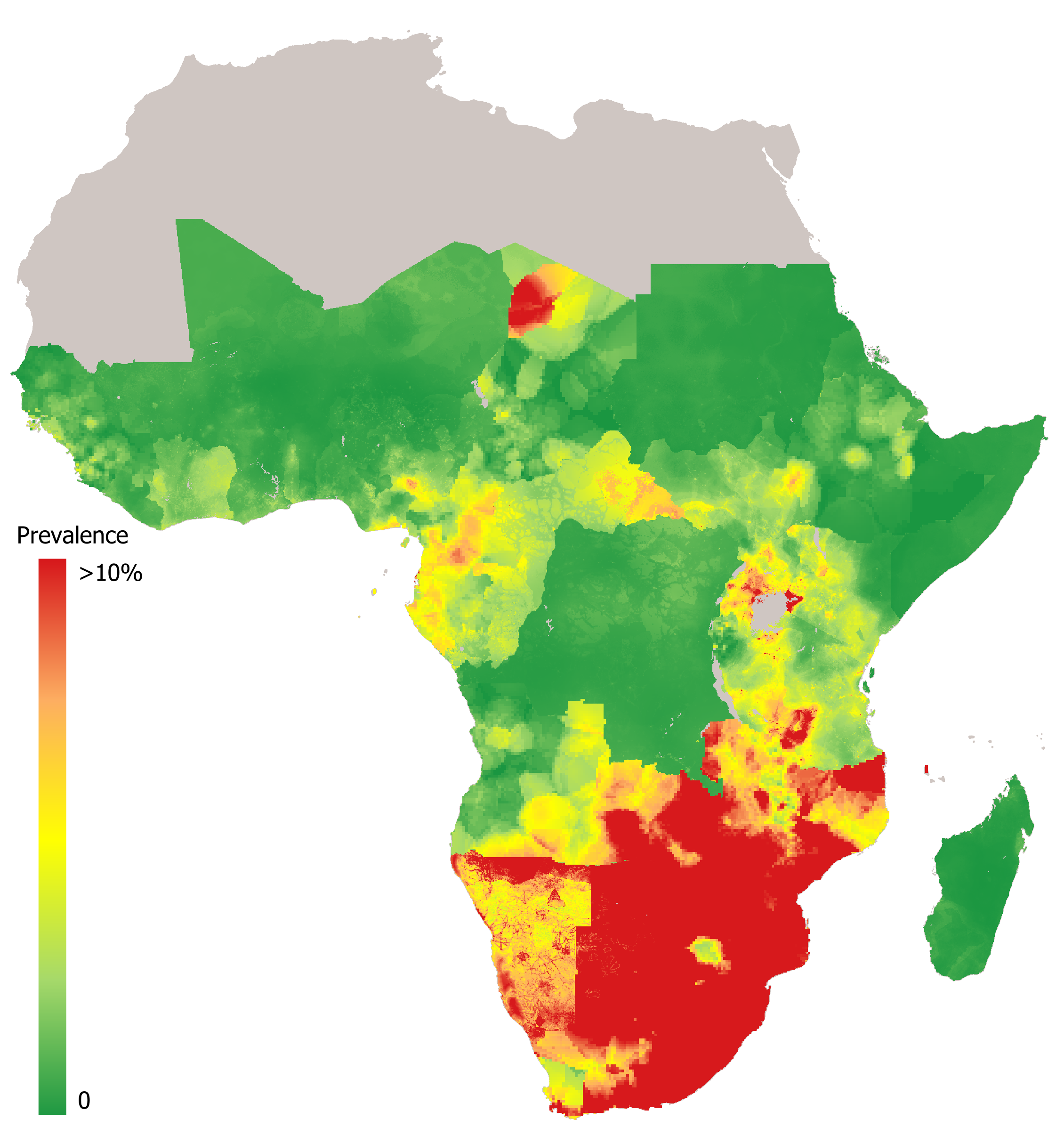


Supplementary Figure 6. HIV prevalence Interpolation in SSA


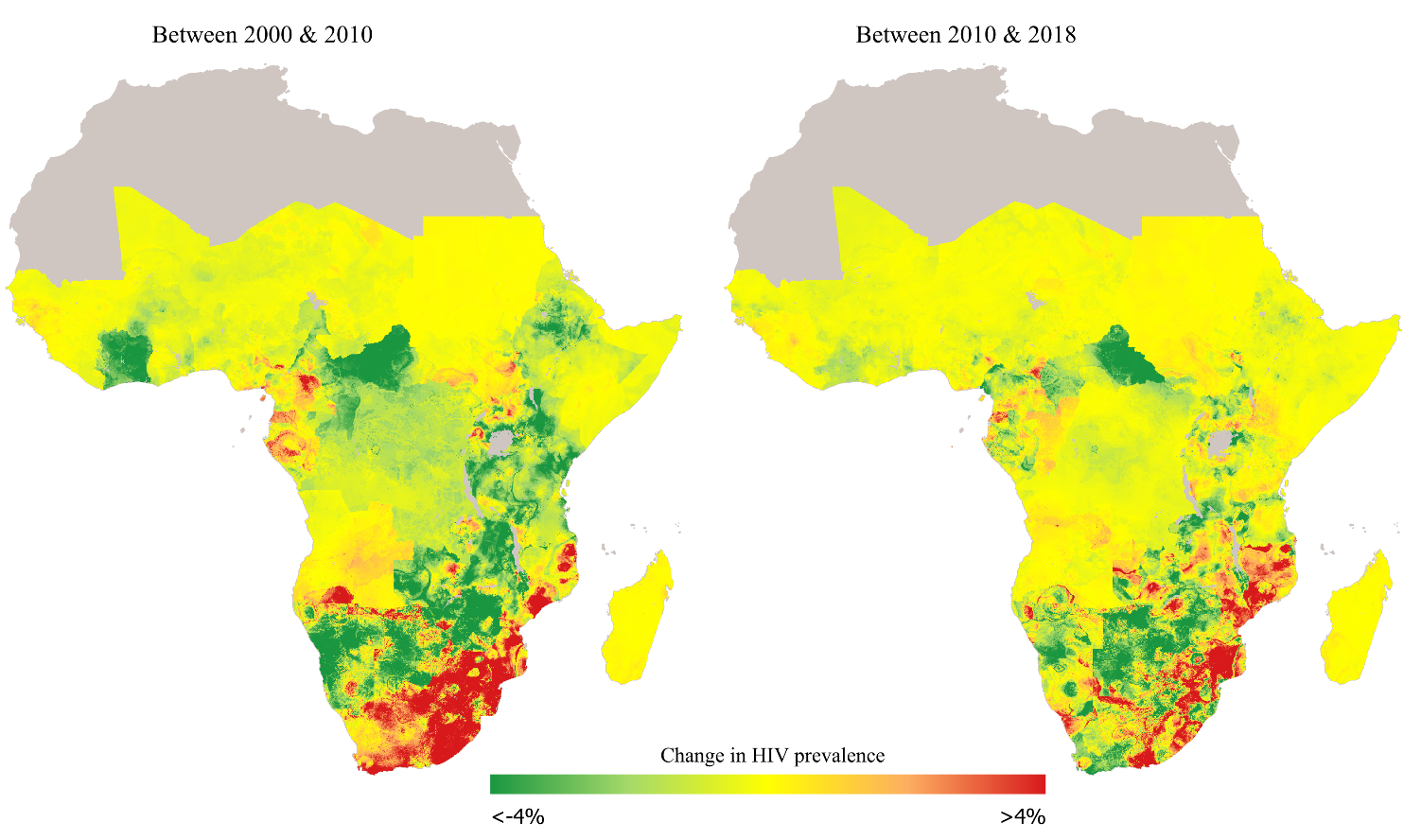


Supplementary Figure 7. Community based change over time. Between the year 2010 and 2000 (left panel), between 2010 and 2018 (right panel)

# **Eigenvalues/variances of principal components and proportion of contribution**

| **Dimension** | **Eigenvalue** | **Percent** | **Cumulative percent** |
| --- | --- | --- | --- |
| Dim.1 | 7.130284 | 26.40846 | 26.40846 |
| Dim.2 | 2.677323 | 9.91601 | 36.32447 |
| Dim.3 | 2.075693 | 7.687752 | 44.01222 |
| Dim.4 | 1.876484 | 6.949941 | 50.96216 |
| Dim.5 | 1.269783 | 4.702899 | 55.66506 |
| Dim.6 | 1.186142 | 4.393118 | 60.05818 |
| Dim.7 | 1.027511 | 3.805595 | 63.86377 |
| Dim.8 | 0.915359 | 3.39022 | 67.25399 |
| Dim.9 | 0.864693 | 3.202568 | 70.45656 |
| Dim.10 | 0.81163 | 3.006035 | 73.4626 |
| Dim.11 | 0.769231 | 2.849004 | 76.3116 |
| Dim.12 | 0.698314 | 2.586348 | 78.89795 |
| Dim.13 | 0.6781 | 2.511482 | 81.40943 |
| Dim.14 | 0.656721 | 2.4323 | 83.84173 |
| Dim.15 | 0.579043 | 2.144603 | 85.98633 |
| Dim.16 | 0.494182 | 1.830305 | 87.81664 |
| Dim.17 | 0.466601 | 1.728153 | 89.54479 |
| Dim.18 | 0.445259 | 1.649106 | 91.1939 |
| Dim.19 | 0.415053 | 1.537235 | 92.73113 |
| Dim.20 | 0.387979 | 1.436958 | 94.16809 |
| Dim.21 | 0.349215 | 1.293389 | 95.46148 |
| Dim.22 | 0.304898 | 1.129252 | 96.59073 |
| Dim.23 | 0.250355 | 0.927241 | 97.51797 |
| Dim.24 | 0.222678 | 0.824732 | 98.34271 |
| Dim.25 | 0.202194 | 0.748869 | 99.09157 |
| Dim.26 | 0.20126 | 0.745406 | 99.83698 |
| Dim.27 | 0.044015 | 0.16302 | 100 |

Supplementary Table 2: Eigenvalues/variances of principal components. Proportion of information and cumulative percent retained by each principal component.

# **Contribution of variables of the selected four principal components**

| **Variable** | **Dim.1** |  | **Variable** | **Dim.2** |  | **Variable** | **Dim.3** |  | **Variable** | **Dim.4** |
| --- | --- | --- | --- | --- | --- | --- | --- | --- | --- | --- |
| Literacy | 8.9 |  | No partner except spouse | 12.65 |  | Circumcised | 19.58 |  | Working status | 15.09 |
| Cohabitation | 7.62 |  | No of sex partner | 11.31 |  | Housing | 10.05 |  | Food insecurity | 14.54 |
| Media exposure | 6.99 |  | PPR/PPP | 10.61 |  | Migration | 9.57 |  | No of sex partner | 10.74 |
| HIV test | 6.77 |  | City | 8.96 |  | Stigma | 8.77 |  | STI | 10.33 |
| Negotiating sex | 6.53 |  | MER | 8.36 |  | HIV test | 8.7 |  | Age at first sex | 8.34 |
| MER | 6.47 |  | GDP | 6.57 |  | HIV mother to child | 6.45 |  | HIV mother to child | 5.05 |
| GDP | 5.74 |  | Distance to disaster | 6.36 |  | No partner except spouse | 6.02 |  | PPR/PPP | 4.32 |
| Stigma | 5.21 |  | Decision making | 6.29 |  | HH wealth | 5.45 |  | No partner except spouse | 4.11 |
| PPR/PPP | 5.16 |  | Time to health facility | 4.77 |  | No of sex partner | 3.91 |  | Migration | 3.54 |
| City | 4.4 |  | Negotiating sex | 4.33 |  | Media exposure | 3.03 |  | Comprehensive knowledge | 3.51 |
| Housing | 3.8 |  | Literacy | 3.46 |  | Working status | 2.7 |  | MER | 3.43 |
| Time to health facility | 3.79 |  | Circumcised | 2.56 |  | Comprehensive knowledge | 2.58 |  | Housing | 3.1 |
| HH wealth | 3.68 |  | Disagreement | 2.35 |  | Time to health facility | 2.37 |  | HH wealth | 2.91 |
| Age at first sex | 3.67 |  | Age at first sex | 1.9 |  | Disagreement | 2.28 |  | Literacy | 2.59 |
| Comprehensive knowledge | 3.4 |  | HIV test | 1.89 |  | Food insecurity | 1.97 |  | Circumcised | 2.38 |
| Decision making | 3.22 |  | Stigma | 1.87 |  | STI | 1.54 |  | Stigma | 2.02 |
| No partner except spouse | 2.79 |  | Housing | 1.42 |  | GDP | 1.35 |  | Years lived | 0.87 |
| Disagreement | 2.61 |  | Cohabitation | 1.15 |  | Distance to disaster | 1 |  | Media exposure | 0.8 |
| Years lived | 2.05 |  | Working status | 1.13 |  | Decision making | 0.86 |  | Cohabitation | 0.5 |
| Distance to disaster | 1.72 |  | Years lived | 0.86 |  | Cohabitation | 0.52 |  | Distance to disaster | 0.41 |
| Circumcised | 1.59 |  | STI | 0.4 |  | Age at first sex | 0.4 |  | GDP | 0.4 |
| HIV mother to child | 1.45 |  | HH wealth | 0.36 |  | PPR/PPP | 0.34 |  | City | 0.34 |
| No of sex partner | 1.37 |  | Migration | 0.28 |  | MER | 0.2 |  | Decision making | 0.32 |
| Food insecurity | 0.67 |  | Comprehensive knowledge | 0.07 |  | Negotiating sex | 0.16 |  | Time to health facility | 0.25 |
| Working status | 0.25 |  | Food insecurity | 0.04 |  | Years lived | 0.1 |  | Negotiating sex | 0.1 |
| STI | 0.16 |  | Media exposure | 0.03 |  | City | 0.06 |  | Disagreement | 0.01 |
| Migration | 0.02 |  | HIV mother to child | 0.01 |  | Literacy | 0.04 |  | HIV test | 0.01 |

Supplementary Table 3: the list of contribution of variables of the selected four principal components. Proportion of information of variables retained by four principal component.


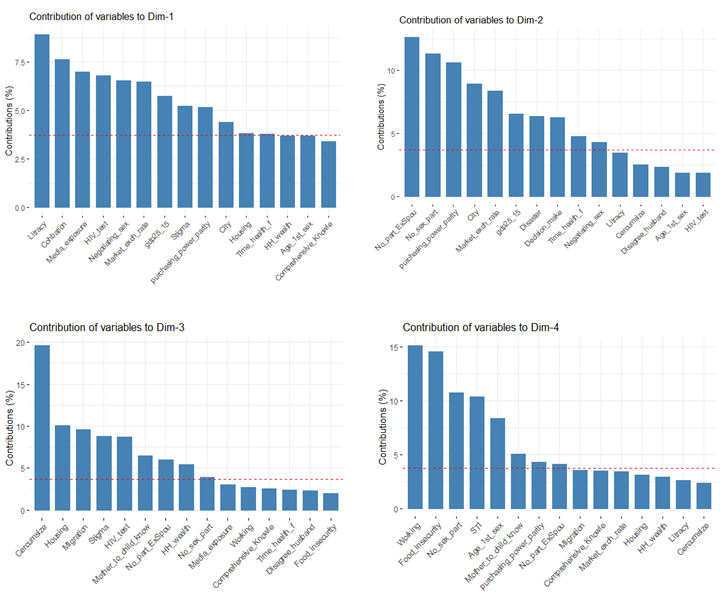


Supplementary Figure 8. Proportion of variables contribution for the four most important PCs
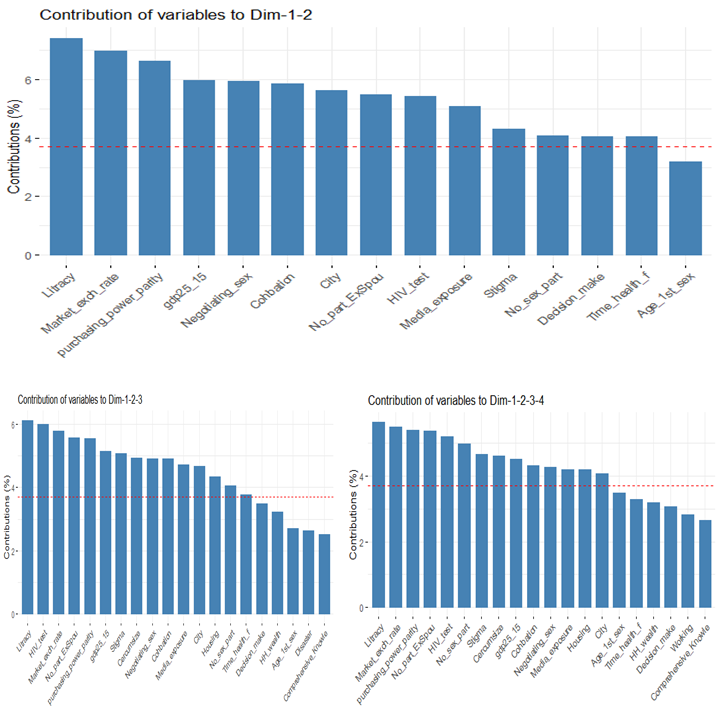


Supplementary Figure 9. Proportion of variables contribution for the combination of two or more important PCs

| **Variable** | **Dim.1** |  | **Variable** | **Dim.2** |  | **Variable** | **Dim.3** |  | **Variable** | **Dim.4** |
| --- | --- | --- | --- | --- | --- | --- | --- | --- | --- | --- |
| Literacy | 0.63 |  | No partner except spouse | 0.34 |  | Circumcised | 0.41 |  | Working status | 0.28 |
| Cohabitation | 0.54 |  | No of sex partner | 0.3 |  | Housing | 0.21 |  | Food insecurity | 0.27 |
| Media exposure | 0.5 |  | PPR/PPP | 0.28 |  | Migration | 0.2 |  | No of sex partner | 0.2 |
| HIV test | 0.48 |  | City | 0.24 |  | HIV test | 0.18 |  | STI | 0.19 |
| Negotiating sex | 0.47 |  | MER | 0.22 |  | Stigma | 0.18 |  | Age at first sex | 0.16 |
| MER | 0.46 |  | GDP | 0.18 |  | HIV mother to child | 0.13 |  | HIV mother to child | 0.09 |
| GDP | 0.41 |  | Decision making | 0.17 |  | No partner except spouse | 0.13 |  | No partner except spouse | 0.08 |
| PPR/PPP | 0.37 |  | Distance to disaster | 0.17 |  | HH wealth | 0.11 |  | PPR/PPP | 0.08 |
| Stigma | 0.37 |  | Time to health facility | 0.13 |  | No of sex partner | 0.08 |  | Comprehensive knowledge | 0.07 |
| City | 0.31 |  | Negotiating sex | 0.12 |  | Media exposure | 0.06 |  | Migration | 0.07 |
| Housing | 0.27 |  | Literacy | 0.09 |  | Working status | 0.06 |  | Housing | 0.06 |
| Time to health facility | 0.27 |  | Circumcised | 0.07 |  | Comprehensive knowledge | 0.05 |  | MER | 0.06 |
| Age at first sex | 0.26 |  | Disagreement | 0.06 |  | Disagreement | 0.05 |  | HH wealth | 0.05 |
| HH wealth | 0.26 |  | Age at first sex | 0.05 |  | Time to health facility | 0.05 |  | Literacy | 0.05 |
| Comprehensive knowledge | 0.24 |  | HIV test | 0.05 |  | Food insecurity | 0.04 |  | Circumcised | 0.04 |
| Decision making | 0.23 |  | Stigma | 0.05 |  | GDP | 0.03 |  | Stigma | 0.04 |
| No partner except spouse | 0.2 |  | Housing | 0.04 |  | STI | 0.03 |  | Media exposure | 0.02 |
| Disagreement | 0.19 |  | Cohabitation | 0.03 |  | Decision making | 0.02 |  | Years lived | 0.02 |
| Years lived | 0.15 |  | Working status | 0.03 |  | Distance to disaster | 0.02 |  | City | 0.01 |
| Distance to disaster | 0.12 |  | Years lived | 0.02 |  | Age at first sex | 0.01 |  | Cohabitation | 0.01 |
| Circumcised | 0.11 |  | HH wealth | 0.01 |  | Cohabitation | 0.01 |  | Decision making | 0.01 |
| HIV mother to child | 0.1 |  | Migration | 0.01 |  | PPR/PPP | 0.01 |  | Distance to disaster | 0.01 |
| No of sex partner | 0.1 |  | STI | 0.01 |  | City | 0 |  | GDP | 0.01 |
| Food insecurity | 0.05 |  | Comprehensive knowledge | 0 |  | Literacy | 0 |  | Disagreement | 0 |
| Working status | 0.02 |  | Food insecurity | 0 |  | MER | 0 |  | HIV test | 0 |
| STI | 0.01 |  | Media exposure | 0 |  | Negotiating sex | 0 |  | Negotiating sex | 0 |
| Migration | 0 |  | HIV mother to child | 0 |  | Years lived | 0 |  | Time to health facility | 0 |

Supplementary Table 4: Variables quality of representation of the four most important principal components.


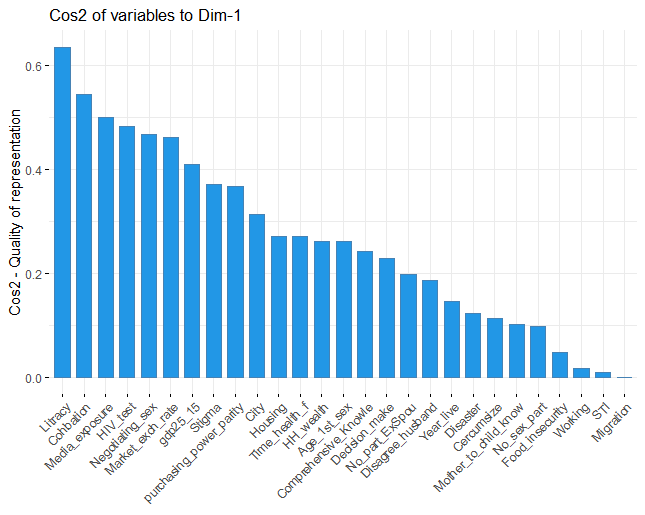


Supplementary Figure 10. Variables quality of representation of the first most important principal component.


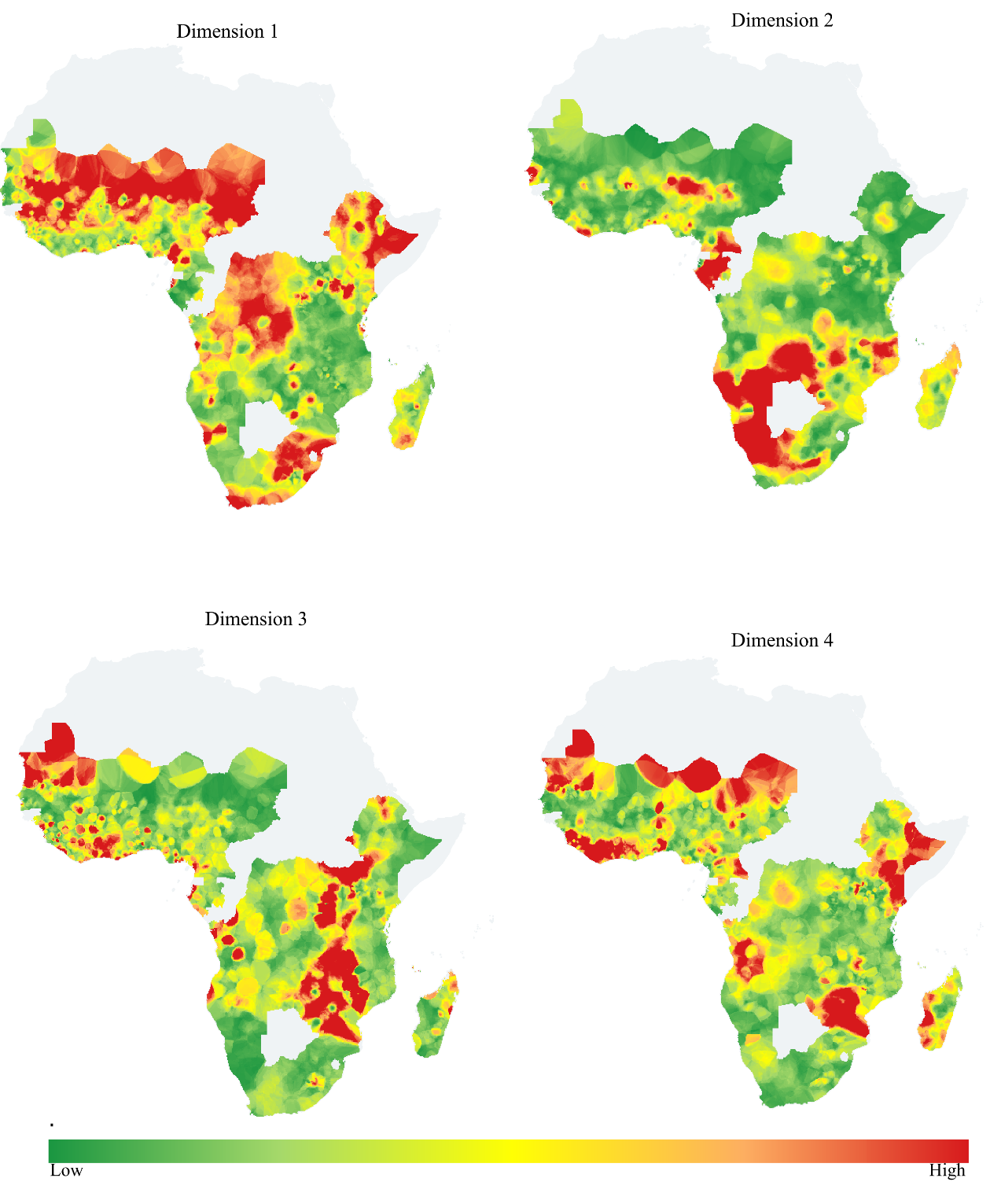


Supplementary Figure 11. Communities’ quality of representation of the four principal components
